# Infection Unit Density as an Index of Infection Potential of Arbuscular Mycorrhizal Fungi

**DOI:** 10.1264/jsme2.ME17098

**Published:** 2018-03-29

**Authors:** Ryo Ohtomo, Yoshihiro Kobae, Sho Morimoto, Norikuni Oka

**Affiliations:** 1 Hokkaido Agricultural Research Center, NARO Hitsujigaoka 1, Toyohira ward, Sapporo, Hokkaido 062–8555 Japan

**Keywords:** arbuscular mycorrhizal (AM) fungi, infection potential, propagule density, infection unit (IU)

## Abstract

The effective use of arbuscular mycorrhizal (AM) fungal function to promote host plant phosphate uptake in agricultural practice requires the accurate quantitative evaluation of AM fungal infection potential in field soil or AM fungal inoculation material. The number of infection units (IUs), intraradical fungal structures derived from single root entries formed after a short cultivation period, may reflect the number of propagules in soil when pot soil is completely permeated by the host root. However, the original IU method, in which all AM propagules in a pot are counted, requires the fine tuning of plant growing conditions and is considered to be laborious. The objective of the present study was to test whether IU density, not the total count of IU, but the number of IUs per unit root length, reflects the density of AM fungal propagules in soil. IU density assessed after 12 d of host plant cultivation and 3,3′-diaminobenzidine (DAB) staining showed a stronger linear correlation with propagule density than the mean infection percentage (MIP). In addition, IU density was affected less by the host plant species than MIP. We suggest that IU density provides a more rapid and reliable quantitation of the propagule density of AM fungi than MIP or the original IU method. Thus, IU density may be a more robust index of AM fungal infection potential for research and practical applications.

Arbuscular mycorrhizal (AM) fungi promote plant growth by facilitating the uptake of nutrients such as phosphorus from soil. Previous studies reported that a sufficient indigenous AM fungal population in soil reduced the amount of phosphate fertilizer conventionally prescribed for AM-host crops (20, 33). Accumulated evidence has demonstrated the beneficial effects of the application of AM fungi in field use. For example, the application of an AM fungal inoculum for field use to improve agricultural yield or for environmental conservation has been examined (21, 25, 26), and the inoculation of AM fungal materials into plant seedlings was shown to increase the survival and growth of transplanted plantlets (12). At the same time, the importance of a pre-evaluation of indigenous AM fungal activity was suggested by a study in which the application of an AM fungal inoculum to welsh onion seedlings compensated for the reduced amount of phosphate fertilizer, but only when the indigenous AM fungal density was not high (29). Therefore, an efficient method to quantify AM fungal infection potential is essential for the effective utilization of AM fungal function in agricultural practice.

Two procedures have been widely used to assess the infection potential of AM fungi: the most probable number (MPN) and mean infection percentage (MIP) (7). In the MIP assay, the colonization intensity of AM fungi is measured after a certain period of bait plant cultivation. Although in the original study on MIP, an index of root colonization was the percentage of the number of 1-cm root segments showing detectable AM fungal colonization (18), the percentage of the root length colonized is now more widely used (9, 10, 16, 23, 27) as a more objective index (17). The MPN method (6), in which test plants are grown in serial dilutions of the inoculum and the propagule density of the original material is statistically calculated from MPN scores, has also been widely used.

As an alternative, Franson and Bethlenfalvay (7) proposed a method to directly count AM fungal propagule numbers in soil based on the number of infection units (IUs), defined as the number of individual fungal structures created by single root entries (termed the infection-unit method, IUM). The main advantage of this method is that the value obtained is a direct count of the propagule rather than an estimate, as in the case of MIP or MPN. Similar to the plate count technique for bacterial enumeration in which each colony is derived from a single bacterial cell, each IU is derived from a single propagule of AM fungi unless secondary infection has not occurred and neighboring IUs are not combined with each other. Using this method, Carrillo-Garcia *et al.* (3) showed that AM propagule densities were significantly lower in plant-free areas as opposed to under plant canopies. Zahka *et al.* (34) reported that the number of IUs in the root systems of assay plants linearly correlated with the proportion of test soil in the growth medium, and also that the colonization level of forest plants more strongly correlated with viable propagule density estimated by the IUM assay, than with soil spore density.

Although IUM is considered to provide a 1:1 correspondence between the numbers of infective propagules and IUs, it is laborious and not frequently used in practice (27). The extensive fine tuning of culture conditions is needed because the root of the test plant must completely permeate the entire growth vessel without crowding in order to guarantee that every AM propagule in soil forms entry points within a relatively short cultivation period (7). This difficulty with original IUM may be avoided by using an IU number standardized to the test plant root length (modified infection unit method, mIUM, Fig. S1). By using IU per root length as an index, Carvalho *et al.* (4) showed that mycelia and/or infected root fragments were a more important AM inoculum than spores in a salt marsh environment. López-García *et al.* (15) also used IU per root length as an index to show that the AM fungal colonization potential of sieved soil was significantly lower than that of natural (undisturbed) soil when soil samples were collected during the plant-growing season.

A stable quantitative relationship between the number of IU per root length and inoculum concentrations is a prerequisite for use as a practical index of inoculum density; however, this has not yet been confirmed. In the present study, we examined the utility of IU density as an index of AM fungal propagule density in soil. Since AM propagule density is a value inherently independent of the plant species used in experiments, we employed four plant species to compare mIUM and MIP in order to establish which is a more appropriate index of AM propagule density. Based on the results obtained showing that mIUM is more strongly dependent on inoculum density and less dependent on host species, we propose the use of IU density per unit root length after a short cultivation period as an index of AM propagule density in soil.

## Materials and Methods

### Fungal inoculum and plant growth media

The arbuscular mycorrhizal fungus inoculum “Glomus R10” (IKGSP1001), which contains *Glomus sp.* R10 as the main inoculant species, was purchased from Idemitsu Kosan (Tokyo, Japan). A single isolate culture of *Claroideoglomus etunicatum* (W.N. Becker & Gerd.) MAFF 520053 was obtained from NARO Genebank (previously MAFF Genebank, http://www.gene.affrc.go.jp/index_en.php) and proliferated using *Lotus japonicus* MG20 as a host. After four months of growth of the host plant and one month of drying, the resultant *C. etunicatum* culture was passed through a 2-mm sieve and stored at 4°C until used.

The plant growth medium used was a 1:1 (w:w) mixture of sterile soil and sand. Soil was collected from an experimental field at the Hokkaido Agricultural Research Center, NARO (Sapporo, Hokkaido, Japan, N 43.01 E 141.41). It was classified as Thapto-upland Wet Andosol (Typic Endoaquands [USDA Soil Taxonomy]) (19). The chemical characterization of the soil indicated a pH (H_2_O) of 5.0, available nitrogen (hot water extractable nitrogen) of 89.6 mg, available phosphate content (Truog) of 143 mg P_2_O_5_, exchangeable K_2_O of 291 mg, and phosphate-absorbing capacity of 11.49 g P_2_O_5_ kg^−1^ soil. Soil was autoclaved twice at 121°C for 20 min with an interval of 1 d between the two autoclaving procedures before use. The commercially available river sand used was washed with tap water to remove fine particles and autoclaved at 121°C for 20 min. Soil and sand were passed through a 2-mm sieve before being added to 50-mL tubes for the mIUM and MIP assays.

### Measuring IU density and MIP

The seeds of the four host plants belonging to three host species: *L. japonicus* (cv. Miyakojima MG20 and cv. Gifu B129), onion (*Allium cepa* cv. Sapporoki), and chive (*Allium schoenoprasum*, Takii Seed, Kyoto, Japan), were germinated on wet paper in a plant growth chamber (MLR351; SANYO, Tokyo, Japan) at 25°C/23°C under a 16-h day/8-h night cycle for 3, 5, and 7 d, respectively. The seeds of *L. japonicus* were pretreated with concentrated H_2_SO_4_ to break down hard seeds. Plastic pots (500 mL) or light-shielded polypropylene test tubes (50 mL) (Sarstedt K.K, Tokyo, Japan) with bottom holes covered with glass microfiber filters (GF/A; Whatman, Maidstone, UK) were used for plant cultivation. The pots and tubes were filled with growth media mixed with the indicated amount of AM fungal inoculum material and were watered well before transplanting the uniformly grown seedlings of each host species.

In the IU density measurement of Glomus R10 material, three independent experiments were performed. In Experiment I, pots at a given inoculum concentration (1, 5, and 25 g pot^−1^) were transplanted with five MG20 seedlings, and the IU density of each plantlet was measured independently (for five independent measures in each pot). In Experiments II (0, 5, 10, and 20 g pot^−1^) and III (0, 5, 10, 15, 20, 25, and 30 g pot^−1^) (a reproducibility trial), three pots were transplanted with three seedlings each of MG20 at a given inoculum concentration, and IU density and root colonization were measured using the whole root system of one pot (for three independent replicates for each inoculum concentration). In comparisons of the mIUM and MIP measurements of the *C. etunicatum* inoculum with different hosts, four 50-mL tubes for each host group were transplanted with two seedlings each at a given inoculum concentration (for four independent replicates at each inoculum in each host).

Pots and tubes were incubated in the same growth chamber described above (MLR-351; SANYO). Plants cultured for 12 d were used for mIUM and 12-d MIP measurements, while plants cultured for 6 weeks were used for 6-week MIP measurements. Plants were covered with plastic wrap to prevent surface drying for the 1^st^ week. After the 2^nd^ week, cultures for 6-week MIP measurements received watering at intervals of 2–3 d, while those for mIUM and 12-d MIP measurements received no additional watering until harvesting. At harvesting, the entire root system in each pot or tube was recovered, washed with tap water to remove soil particles, and soaked in 50% ethanol overnight or longer for fixation. Fine blots were removed from roots by 1 min of sonication in a water bath during the fixation step.

The roots of the 12-d culture were stained with 3,3′-diaminobenzidine (DAB), as described previously (14), with slight modifications. Briefly, roots were transferred into 12-well microplates, cleared in 2 mL of 10% KOH at 80°C for 3 h, acidified by adding 1 mL of 3 mol L^−1^ HCl, washed extensively with water, and stored in phosphate-buffered saline (PBS, pH 7.5). Fungal structures were labeled with wheat germ agglutinin (WGA)-conjugated horseradish peroxidase (WGA-HRP; VECTOR LABORATORIES, Burlingame, CA, USA) and detected with DAB. The concentration of WGAHRP used was 50% that of the original method (14) by a dilution in 50% glycerol, 10 mM Tris-HCl (pH 7.5) solution. This diluted WGA-HRP stock was stable for at least several months at −20°C. DAB-stained roots were spread in shallow PBS on the bottom of 60-mm ø plastic dishes, and covered with grid-lined nitrocellulose membrane filters (ADVANTEC, Tokyo, Japan) with the lined surface facing down against the root. After excess liquid was removed, the plate was turned upside down and the root was inspected from the bottom of the dish (Fig. S2A) using a stereoscopic microscope (SMZ-U; Nikon, Tokyo, Japan) at 30× magnification in order to record the number of IUs (N_IU_) and number of intersections of roots with grid lines (N). A clump of the AM fungal structure stained dark brown with arbuscules was regarded as a single IU irrespective of size (Fig. S2B, C, and D). Total root length was calculated according to Tennant (30) as:

(Total root length,m)=πNx/4,

where N is the number of intersections of the root and grid line, and *x* is the interval of grid lines (0.003 m). IU density (m^−1^) was then calculated as the number of IUs (N_IU_) divided by root length (m), namely:

$$(\text{IU density},{\text{m}}^{-1})={\text{N}}_{\text{IU}}/(\text{Total root length})=4{\text{N}}_{\text{IU}}/\pi \text{Nx.}$$

The proportion of the colonized root length at 12 d (12-d MIP) was measured using the same sample with IU density measurements, and calculated as a ratio of AM fungal-positive root-gridline intersections (M) against all root-grid line intersections (N). This 12-d MIP was only calculated as a reference because there were less than 100 observation points (*i.e.* root-grid line intersections, N) in some samples.

(12-d MIP)=M/N

The difference between IU counting (for mIUM) and colonization intensity measurements (for MIP) is shown in Fig. S3.

Regarding MIP measurements of 6-week cultures (6-week MIP), the root was fragmented into pieces of 2–3 cm and stained with trypan blue as previously described (14). In brief, the root fragment was cleared and acidified as above and stained in 1 mL of 0.05% (w/v) trypan blue/lactic acid at 80°C for 1 h. After removing excess dye by immersing the stained root in lactoglycerol overnight or longer, randomly selected root fragments were mounted on slide glasses and mycorrhizal structures were detected under a light microscope (ECLIPSE E600; Nikon) at 100× magnification at more than 150 points. MIP was calculated as a ratio of AM-positive positions (M′) among all observed points (N′) as:

$$(6\text{-week MIP})={\text{M}}^{\prime }/{\text{N}}^{\prime }.$$

### Statistical analysis

Treatment group means were compared by Tukey’s honestly significant difference (HSD) test with a significance level of 5%. The effects of the inoculum amount on IU density among reproducibility trials were evaluated with an analysis of covariance (ANCOVA), and the effects of the inoculum amount on the root length or IU density of different host species were evaluated with an analysis of variance (ANOVA). Pearson’s product moment correlation coefficients (r) and their *P*-values were calculated in order to evaluate linearity. All statistical analyses were performed using JMP software (ver. 12.2.0; SAS Institute, USA).

## Results

### IU density was proportional to inoculum density

**Fig. 1:**
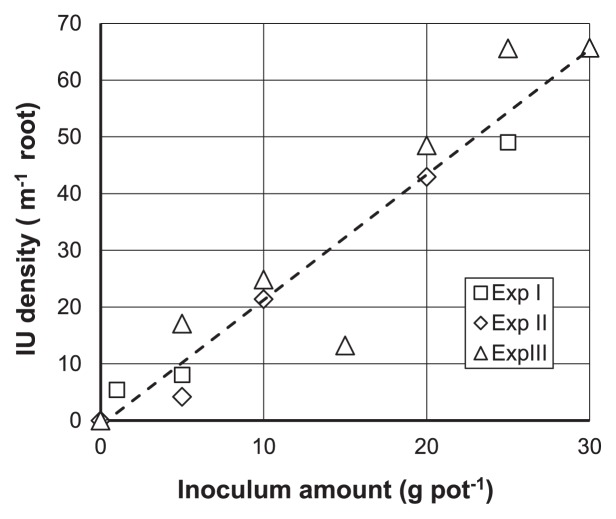
Linear relationship between IU density and inoculum concentration *Lotus japonicus* MG20 seedlings were grown in 500-mL pots inoculated with the indicated amount of Glomus R10 material, and IU density was assessed 12 d after transplant. Experiments were repeated three times (Exp. I, rectangles; Exp. II, diamonds; and Exp. III, triangles) and replicated 5 times (Exp. I) or 3 times (Exp. II and III) for each inoculum concentration (see text for details). Points indicate the average at each dilution. The regression line was calculated using the data from all three experiments.

In order to confirm that IU density provides a reproducible and quantitative index of the propagule density of AM fungi, we measured IU density on the roots of *L. japonicus* MG20 in triplicate cultures with sterile growth medium (sand plus soil) inoculated with different Glomus R10 concentrations. In Experiment I, the IUs of five seedlings per pot were independently assessed at each inoculum dilution. The mean (±SD) root length of each plantlet was 114±21 mm and was not affected by the inoculum density (Tukey’s HSD test). In Experiments II and III, the whole root system in each pot was used for a single IU measurement, with three pots per inoculum concentration. The mean±SD total root length for all three plantlets per pot was 277±54 mm in Experiment II and 330±105 mm in Experiment III. The IU density increased linearly with the inoculum concentration (Fig. 1). Furthermore, IU density values for Experiments I-III were similar and appeared to cluster equally with the overall regression line. The regression equation calculated using results from all three experiments was:

$$\left(\text{IU density},{\text{m}}^{-1}\;\text{root})=2.21\times (\text{Inoculum amount},\text{g }{\text{pot}}^{-1})-0.83\;({\text{R}}^{2}=0.91,P<0.0001\right)$$

The two-way ANCOVA examining the effects of experimental series (I–III) and inoculum concentrations on IU density revealed no significant series × inoculum concentration interaction (*P*=0.57), indicating that the effects of the inoculum concentration on IU density did not significantly differ among the experiments (*i.e.*, it was reproducible). In contrast, the proportion of the colonized root length assessed using the same samples after 12 d (12-d MIP) yielded a weaker linear relationship (Fig. S4) (R^2^=0.79, *P*<0.0001).

### IU density was affected less by host plant species than MIP

**Fig. 2:**
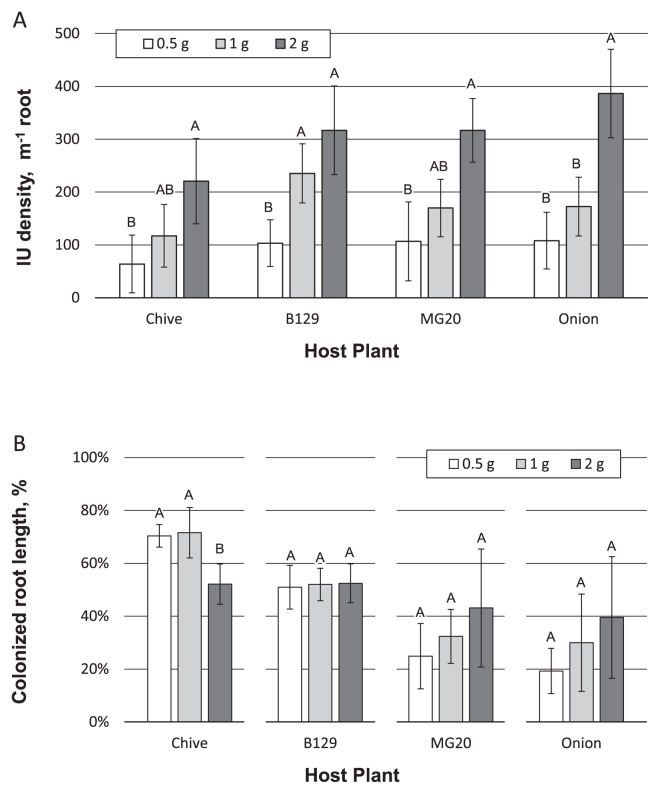
Dependency on host plant species of IU density and MIP Four host plants were used to measure the infection potential of media inoculated with *Claroideoglomus etunicatum* by the IU density (A) or MIP (B) method. Two uniformly grown seedlings of each host were transplanted into each 50-mL tube, which were inoculated with the indicated amount of the *C. etunicatum* culture (0.5 g, white bars; 1 g, light gray bars; 2 g, dark gray bars), and IU density and MIP were then assessed 12 d and 6 weeks, respectively, after transplanting. Bars and error bars indicate averages and standard deviations of 4 replicate cultivations. Bars marked with the same letter show no significant difference within each host plant group at a significance level of 5%.

In order to evaluate the effects of host plant species on AM infection potential measurements using IU density and MIP, *L. japonicus* (MG20 or B129), onion, and chive were grown in separate 50-mL tubes inoculated with different *C. etunicatum* concentrations. The two-way ANOVA indicated that root length was not affected by inoculum density (*P*=0.79), but significantly differed among host plants (*P*<0.0001). The mean root length per tube was 290±7 mm for *L. japonicus* MG20, 106±3 mm for *L. japonicus* B129, 330±4 mm for onion, and 362±10 mm for chive on day 12 after transplant. The mean root length of B129 was significantly shorter than those of the other hosts (*P*<0.0001, by Tukey’s HSD test), while no significant differences were observed in root lengths among those of the other three. The two-way ANOVA indicated that IU density was significantly affected by the inoculum concentration (*P*=1.58×10^−10^) and host species (*P*=0.006). The IU density of chive was significantly smaller than those of B129 (*P*=0.014 by Tukey’s HSD test) and onion (*P*=0.01 by Tukey’s HSD test) across the range of inoculum concentrations, while IU density did not significantly differ among B129, MG20, and onion at the same inoculum concentration. As shown in Fig. 2A, the 0.5-g and 1.0-g *C. etunicatum* inocula in 50-mL tubes formed approximately 100 and 200 IUs per root of *L. japonicus* (B129 or MG20) or onion. Similar to Glomus R10, strong linearity was observed between *C. etunicatum* concentrations and IU densities for chive (*r*=0.75, *P*=0.0047), B129 (*r*=0.80 and *P*=0.0017), MG20 (*r*=0.84, *P*=0.0005), and onion (*r*=0.90, *P*<0.0001).

The proportion of the colonized root length assessed using the same samples after 12 d of cultivation (12-d MIP) showed similar results to IU density, although differences among host plant species were larger and linearity was weaker (chive: *r*=0.67, *P*=0.018; B129: *r*=0.69, *P*=0.012; MG20: *r*=0.81, *P*=0.0011; onion: *r*=0.63, *P*=0.028) (Fig. S5). MIP assessed 6 weeks after transplant exhibited greater dependence on host plant species (Fig. 2B). The two-way ANOVA indicated that the MIP value was significantly affected by the host plant species (*P*=1.77×10^−7^), but not by the inoculum density (*P*=0.43). When 0.5 g of the inoculum was used, for example, MIP values ranged between 20% for onion and more than 70% for chive. Moreover, not all species exhibited a proportional increase in MIP with inoculum concentrations. Although MIP measured with onion or *L. japonicus* MG20 increased with the inoculum density, the values for B129 remained largely unchanged, while MIP values for chive decreased when the inoculum density was greater than 1 g in each tube.

## Discussion

### IU density was effective for evaluating AM fungal infection potential

In the present study, we demonstrated that the density of AM fungal IUs that formed on test plant roots (number of IUs per root length, m^−1^) is a reliable index of propagule concentrations in soil. IU density strongly correlated with AM fungal propagule density in soil over repeated measurements (Fig. 1). Moreover, IU density exhibited several advantages over conventional MIP measurements. IU density was less dependent on host plant species than MIP (Fig. 2A vs. 2B). Since AM propagule density is an inherently independent value to plant species, mIUM is considered to more directly correlate with inoculum density than MIP. Furthermore, IU density was assessed after a shorter cultivation period than MIP (12 d vs. 6 weeks), while still showing a stronger linear association with inoculum concentrations for 4 different host plant species. Although MIP values after a shorter cultivation period (12-d MIP) showed a stronger correlation with inoculum concentrations than that after 6 weeks of cultivation, linearity with inoculum concentrations was weaker than with IU density measures (Fig. S4) and showed greater dependence on host plant species (Fig. S5). Thus, we propose the use of IU density as an index of AM fungal infection potential (modified IUM, mIUM).

The mIUM measurement proposed here counts the number of small fungal structures that have just started to infect plant roots using DAB staining. While fungal structures are also detectable by conventional trypan blue staining, a high background and the fragility of the stained roots preclude IU measurements. A high background makes it difficult to detect small IUs as shown in Fig. S2E. Furthermore, difficulties are associated with keeping fragile trypan blue-stained roots intact (14); however, in the present study, even long roots derived from individual plants were kept intact in order to prevent the separation of IUs between different fragments. Thus, the DAB staining method of Kobae and Ohtomo (14) has made IU counting practical by reducing background staining and preserving intact tissue structures during the staining procedure.

Original IUM requires growth vessels to be completely permeated by the test plant root without substantial crowding in order to guarantee contact with every AM propagule in the container, thereby necessitating the laborious fine tuning of growth conditions. In the case of mIUM, complete permeation of the root is not necessary. In contrast, too much root will decrease IU density because the number of propagules is limited. In the present assay, a 50-mL growth tube provided a sufficient growth space for an approximately 30-cm root in order to yield reproducible results among different plant species.

Although the MPN method is also widely used to evaluate AM propagule density, it was not used here due to several theoretical limitations. Two conditions must be satisfied for the proper application of MPN methods: 1) propagules need to be randomly distributed in the diluent; and 2) every pot with a single propagule needs to yield a positive signal (28). These conditions appear unlikely for AM fungal propagule enumeration, as discussed by Wilson and Trinick (32). A random distribution in diluents appears to be difficult, particularly at the highest dilution, because AM spores are often connected to hyphae or remain attached to fine openings in soil particles. Furthermore, the positive detection of a single propagule in a pot requires the entire pot space to be filled with the host roots and the whole root sample to be investigated for infection. Daniels *et al.* (5) previously reported that infection potential assessed by MPN per AM spore number differed among fungal species, but did not correlate with spore germination capacity. According to Khan (13), MPN values did not correlate well with the inoculum dilution; however, the spore densities of the same samples were pro- portional to the dilution. Feldmann and Idczak (6) recommended MPN for evaluating the infectivity of an AM fungal inoculum because mycelia as well as spores are an important source of root colonization. However, they also found that AM fungi had sporulated inside the cavities of clay particles, which may disrupt the even distribution of individual propagules required to apply the MPN technique. Ademan (1) demonstrated that the inoculum and soil diluent need to have the same origin for the maximum expression of MPN; however, this condition has rarely been considered in subsequent studies. Considering these drawbacks, one of the first studies on the MPN enumeration of AM fungi (22) stated that the accuracy of the method needs to be validated in future experiments and that its use may be restricted to special studies because it is time consuming to obtain results with acceptable variability.

### Short growth time for mIUM

In the present study, a short growth period of 12 d was applied to mIUM. A longer growth period may weaken the relationship with inoculum concentrations due to secondary infection or the coalescence of neighboring entry points (7). Carvalho *et al.* (4) found that a longer growth period decreased the apparent IU value at a given inoculum concentration possibly because of the coalescence of many entry points. The growth time used for MIP measurements markedly differs across studies (*e.g.*, 30 d [18, 23], 7 weeks [16], 60 d [10], and 14 weeks [9]). A shorter growth period is generally considered to more accurately reflect the original propagule density in soil because prolonged growth may increase secondary infection and fungal growth within roots (18). Martinez and Johnson (16) reported that increasing the spore density of soil two- to eight-fold did not induce higher root colonization using the MIP assay, and this may have been because the growth period in their MIP assay was too long. This effect was also observed in the present study; 12-d MIP (Fig. S5) showed a stronger correlation with inoculum density than 6-week MIP (Fig. 2B). However, it is important to note that some AM fungal species with deep dormancy (8, 31) may not be detected using a short cultivation period. In these cases, a breaking dormancy procedure (11, 24, 25) is needed for accurate quantification by mIUM or MIP with a short cultivation period.

### mIUM and MIP are distinct indices

The MIP value was more sensitive to the host plant species, particularly in the case of a long growth period for test plants. When chive or *L. japonicus* B129 was used as a host, MIP values assessed 6 weeks after transplant did not reflect the original AM fungal propagule density in soil; however, the percent root colonization at 12 d showed similar results to those for IU density. Similar findings were reported by Bharadwaj *et al.* (2); the number of AM fungal entry points correlated with the % of infected root segments measured at 30 d, but not with that at 70 d.

These observations do not necessarily mean that the MIP method is inappropriate for AM fungal propagule quantification in all cases. The spread of infection is affected not only by inoculum density, but also by other factors such as the affinity of the host plant for the fungal species, soil chemical properties, and cultivation conditions. We consider the MIP measure to be a more integrative index of AM fungal activity than mIUM, in which not only AM propagule density, but also other factors are taken into account. In contrast, mIUM may be an index that more closely correlates with AM propagule density than MIP. A high IU density does not guarantee the extensive spread of an infection area afterwards because high propagule density may be a necessary, but not sufficient condition for a high infection rate.

## Conclusion

The application of mIUM to measure the AM infection potential of natural soil harboring mixtures of different AM fungal species appears to be practically important. Our preliminary study indicated that IU density measured using soil collected from AM-host plant cultivated fields was larger than that from AM non-host cultured fields, which was consistent with findings indicating that previous cropping with AM-host plants increased AM fungal infection potential (20, 33). Further studies are needed in order to confirm that mIUM is a robust index of AM infection potential, and that it is applicable to comparing infection potentials among different experiments. We are also interested in investigating whether mIUM is effective for predicting the actual AM infection rate under different environmental conditions.

In summary, mIUM provides a direct count of the number of AM propagules colonized per unit length of a root, and strongly correlates with soil AM fungal infection activity. This measure also appears to be less dependent on the host plant species than the conventional MIP method. Therefore, IU density may be a more general and practical index of AM fungal propagule density than the MIP or MPN method.

## Supplementary Material
