# Supplementary material for: Infection Unit Density as an Index of Infection Potential of Arbuscular Mycorrhizal Fungi
Source: Microbes Environ. 2018 Mar 29;33(1):34–9. doi: 10.1264/jsme2.ME17098 (PMC5877340; doi:10.1264/jsme2.ME17098)
Supplement: Supplementary file 1 [file 33_34_s1.pdf]

Supplementary Figures

Fig. S1. Diagrams showing difference between original and modified infection unit (IU) methods

Pot, propagules of arbuscular mycorrhizal (AM) fungi and test plant root are shown with trapezoids, red dots and curved brown lines, respectively. A: In the original infection unit method (IUM), entire space of the pot should be permeated by the test plant root so that every AM propagules establish colonization. Total number of IU is used as an index. B: In the modified IUM (mIUM), the plant root is not permeated, and not all propagules establish colonization. Density of IU is used as an index of propagule density.

Fig. S2. Infection unit (IU) density measurement

The DAB-stained test-plant roots were spread on the bottom of 60-mm ø plastic dishes, covered with grid-lined nitrocellulose membrane filters, and inspected from bottom of the dish (A). In this picture, position of IUs was marked with red ink. IUs were stained dark brown on yellowish root, and different sizes of IU were observed (B-D). Bars in panel B-D indicate 0.5 mm.

Fig. S3. Diagrams showing difference between infection unit (IU) counting and infection rate measurement.

Gray zone in each panel indicates root, and thick lines within the gray zone are IUs. Thin solid lines are gridlines for colonization rate determination. Every root in each panel has 2 IUs although the number of intersections of AM-positive root and gridline is 0, 1, 1, and 2 in panel A, B, C, and D, respectively.

Fig. S4. Correlation between 12-day MIP value and inoculation concentration

MIP measures of AM colonization of the sample used in Fig. 1 are shown. *Lotus japonicus* MG20 was grown in 500 mL pots inoculated with the indicated amount of Glomus R10 material, and AM root colonization was measured 12 days after transplant. Experiments were repeated three times (Exp. I, rectangles; Exp. II, diamonds; and Exp. III, triangles) with 5 (Exp. I) or 3 replicates (Exp. II and III) at each inoculum concentration (see text for detail). Regression line is calculated using the data from all three experiments.

Fig. S5. 12-day MIP measures with different host species.

AM colonization of four host plants which were used for IU density measurement (Fig. 2A) as measured by 12-day MIP. Two uniformly grown seedlings of each host were transplanted to each 50 mL tube

inoculated with the indicated amount of *C. etunicatum* culture (0.5 g, white bars; 1 g, light gray bars; 2 g, dark gray bars). Bars and error bars indicate averages and standard deviations of 3 replicate cultivations. Bars marked with the same letter show no significant difference within each host plant group at a significance level of 5%.

Fig. S1

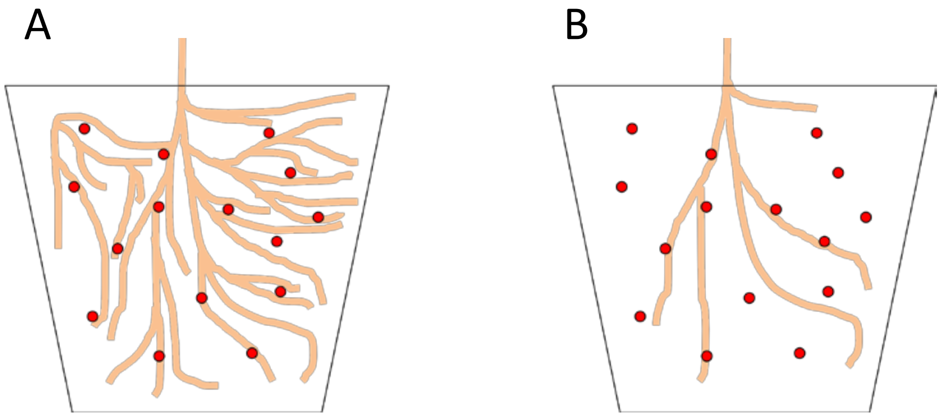

Fig. S2

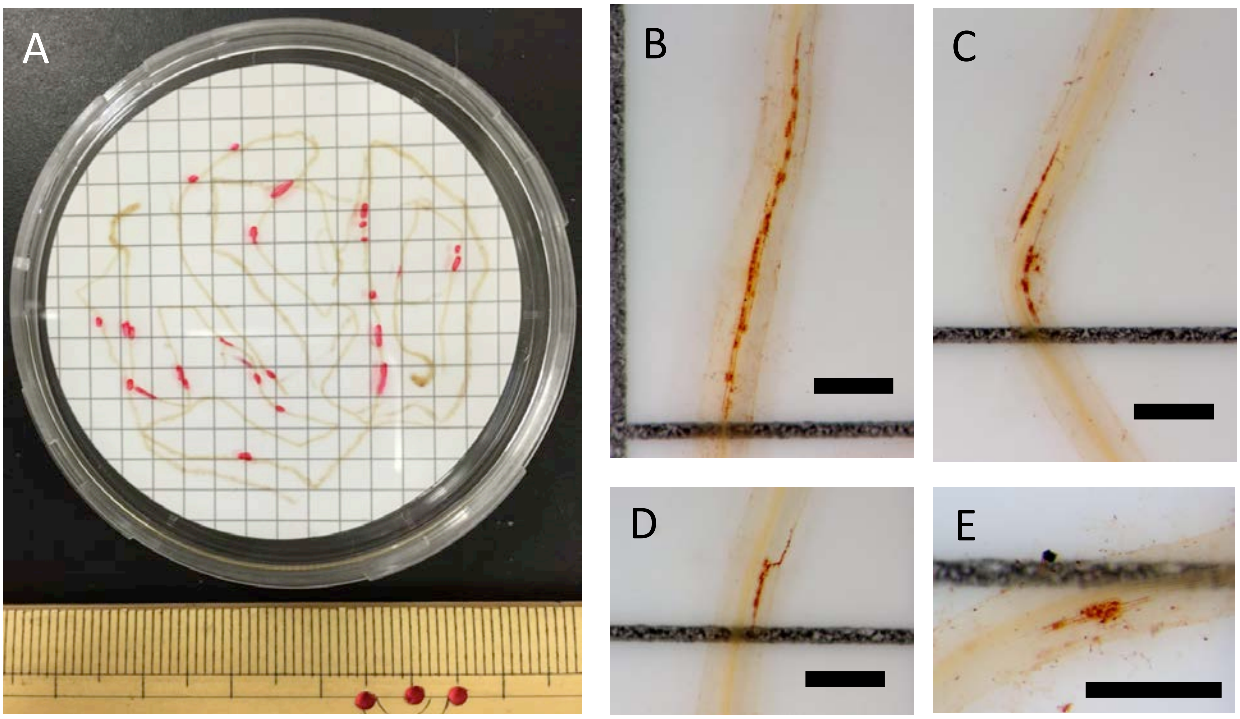

Fig. S3

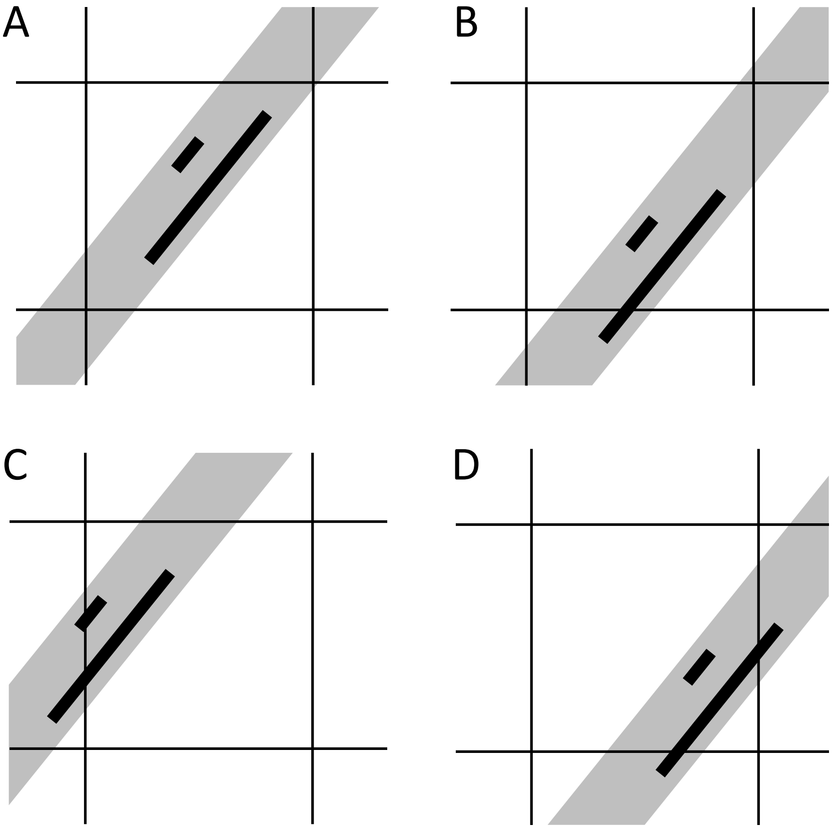
A
B
C
D

Fig. S4

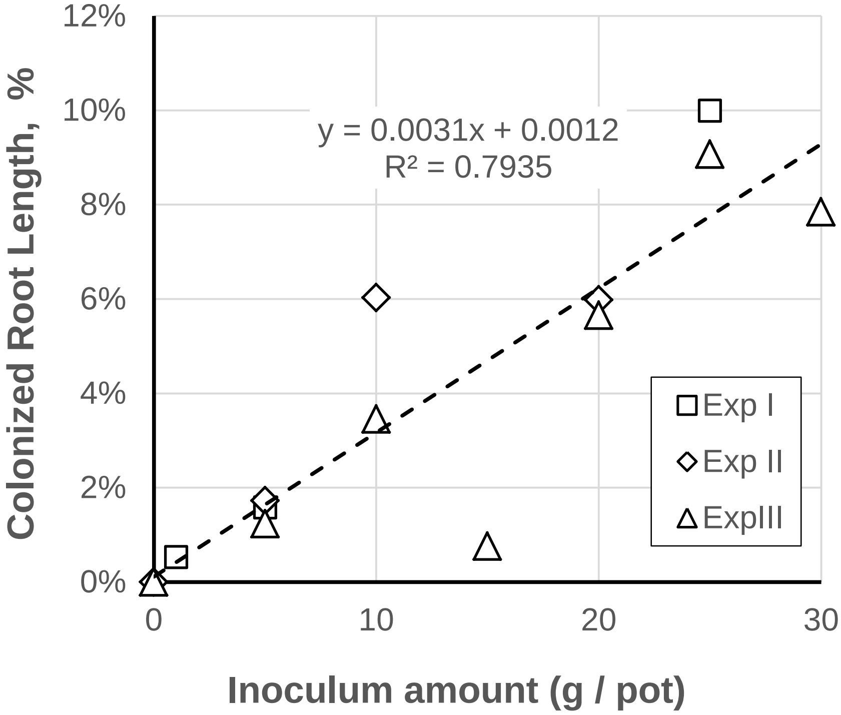
12%
10%
8%
6%
4%
2%
0%
0
10
20
30
y = 0.0031x + 0.0012
R² = 0.7935
Exp I
Exp II
ExpIII
Colonized Root Length, %
Inoculum amount (g / pot)

Fig. S5

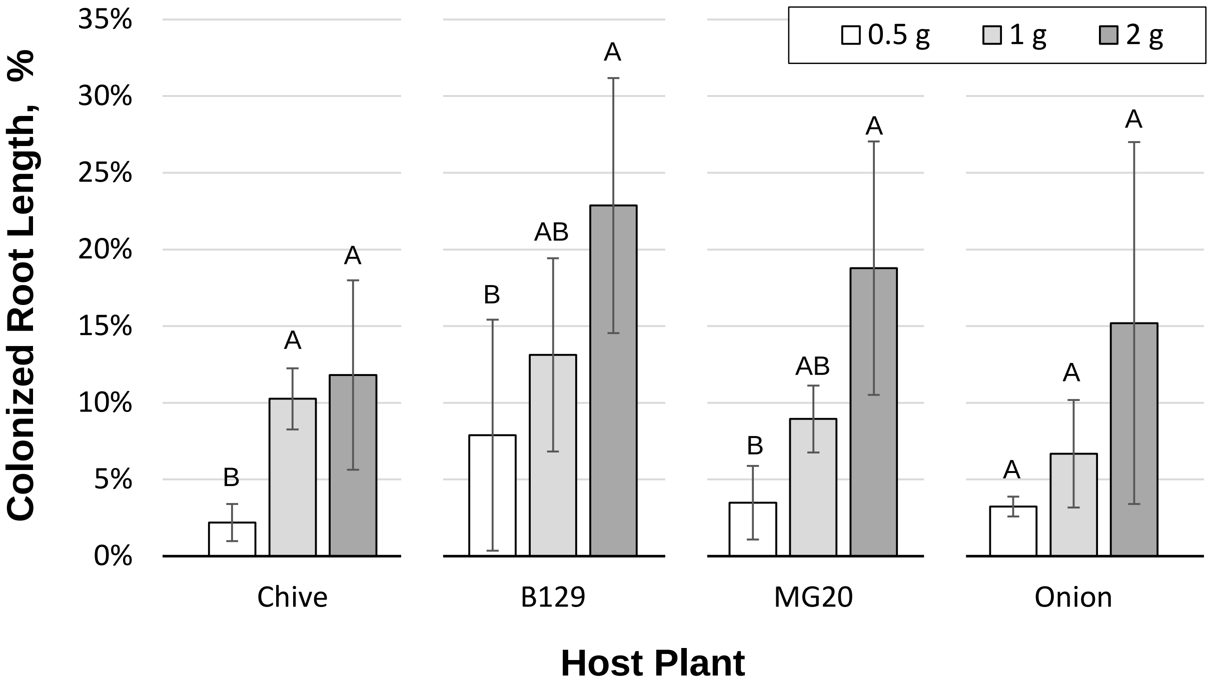

0.5 g
1 g
2 g
Colonized Root Length, %
35%
30%
25%
20%
15%
10%
5%
0%
B
A
A
B
AB
A
B
AB
A
A
A
A
Chive
B129
MG20
Onion
Host Plant
